# Genomotyping of *Coxiella burnetii* Using Microarrays Reveals a Conserved Genomotype for Hard Tick Isolates

**DOI:** 10.1371/journal.pone.0025781

**Published:** 2011-10-25

**Authors:** Quentin Leroy, Fabrice Armougom, Pascal Barbry, Didier Raoult

**Affiliations:** 1 Unité de Recherche en maladies Infectieuses et Tropicales Emergentes (URMITE), CNRS-IRD, UMR 6236, Faculté de Médecine, Université de la Méditerranée, Marseille, France; 2 Institut de Pharmacologie Moléculaire et Cellulaire (IPMC), UMR 6079 CNRS-UNSA, Sophia Antipolis, France; East Carolina University School of Medicine, United States of America

## Abstract

*C. burnetii* is a Gram-negative intracellular Y-proteobacteria that causes the zoonotic disease Q fever. Q fever can manifest as an acute or chronic illness. Different typing methods have been previously developed to classify *C. burnetii* isolates to explore its pathogenicity. Here, we report a comprehensive genomotyping method based on the presence or absence of genes using microarrays. The genomotyping method was then tested in 52 isolates obtained from different geographic areas, different hosts and patients with different clinical manifestations. The analysis revealed the presence of 10 genomotypes organized into 3 groups, with a topology congruent with that obtained through multi-spacer typing. We also found that only 4 genomotypes were specifically associated with acute Q fever, whereas all of the genomotypes could be associated to chronic human infection. Serendipitously, the genomotyping results revealed that all hard tick isolates, including the Nine Mile strain, belong to the same genomotype.

## Introduction

*C. burnetii* is a Gram-negative intracellular Y-proteobacteria that causes Q fever, which is a zoonotic disease with a worldwide distribution [1]. Q fever can manifest as an acute or chronic illness. Acute Q fever is typically a self-limiting febrile illness during which pneumonia or hepatitis can occur, whereas chronic Q fever is a severe illness in which patients can present endocarditis, vascular infection, osteomyelitis and chronic hepatitis [1]. The major route of contamination with *C. burnetii* is aerosol. *C. burnetii* displays antigenic variation in its lipopolysaccharides (LPS) [2]. Phase I is highly infectious and corresponds to the natural phase found in animals, including humans and arthropods, whereas phase II is not very infectious, presents truncated LPS and can be obtained after several passages in cell culture or from embryonated eggs [1]. The *C. burnetii* genome was sequenced in 2003, and its size is approximately 2 Mbp, with a plasmid of approximately 38 kbp [3]. Recently, 3 new isolates of this species were sequenced [4].

Analysis of 16S rDNA gene sequencing data has shown that *C. burnetii* strains isolated from a variety of geographical areas and various hosts display considerable genetic homogeneity [5]. Restriction fragment length polymorphism (RFLP) analysis of genomic DNA (gDNA) [6]–[8] and sequence and/or PCR-RFLP analysis [9]–[12] of specific genes reveal genetic diversity between *C. burnetii* isolates. The most extensive survey of *C. burnetii* genetic diversity was reported by Glazunova et al. [13], who used multi-spacer typing (MST) to genotype approximately 150 *C. burnetii* isolates. More recently, a comparative genomic hybridization (CGH) analysis was performed on a collection a 24 strains of *C. burnetii* [14]. The availability of the *C. burnetii* genome sequence allows a rapid assessment of whole-genome sequence variation by using comparative genome hybridization (CGH) on microarrays, allowing the determination of correlations between the genome repertoire and the source of the organisms.

A long controversy related to the virulence of different isolates of *C. burnetii* was resolved recently [13], [15]–[18]. All types of strains can be isolated from chronic infections that are determined more by host factors than by bacterial factors. In contrast, only particular strains have been isolated from acute infections, and the prototype strain, Nine Mile, has been found to cause acute infection at a lower inoculum concentration than the strain Q212, which is found in association with chronic infection. Therefore, there is a difference of the virulence of strains in causing acute infection that is correlated with the genotype, as determined by MST, genomotyping or plasmid typing.

Q fever is currently re-emerging in different areas in Europe, with a major outbreak of Q fever observed in the Netherlands (causing both acute and chronic infections) [19] and in US military personnel in Iraq [20]. These *C. burnetii* outbreaks bring to the forefront the question of bacterial clonality, which could be related to distribution of highly virulent clones. Alternatively, the apparent massive increase in cases of Q fever could be related to improved detection or increased risk of exposure to animal reservoirs [20]. The widespread outbreak that is presently occurring in the Netherlands has been the focus of numerous molecular biology investigations, including one that indicated that a single genotype, or at least a reduction of heterogeneity, was implicated in the outbreak [19], [21]. We had the opportunity to test this genotype using MST methods [13] and found that the putative clone responsive for the outbreak was identical to a strain isolated from an infected sheep vagina in Germany over 10 years ago and several strains isolated from humans in France.

Although these large outbreaks seem to be related to exposure to domestic and wild animals, the role of arthropods in Q fever transmission has to be considered. The role of ticks as vectors and reservoirs has been discussed since 1937. Ticks may be infected by *C. burnetii* during feeding; excrete it *via* feces, saliva and coxal fluid; and transmit it transovarially and transstadially. The reference strain Nine Mile was isolated from a *Dermacentor andersoni* hard tick, and Q fever was initially presumed to be a vector-borne disease [22]–[25]. At present, ticks are rare vectors for transmission of Q fever [26].

In this study, we compared 52 isolates from patients and animals (mammals, bird and ticks) from the *C. burnetii* strain collection housed in our laboratory, including 2 isolates presenting the same MST genotype as the putative epidemic clone from the Netherlands using DNA whole-genome microarrays to perform genomotyping to investigate associations of the gene repertoire, source and clinical information for *C. burnetii.*

## Materials and Methods

### *C. burnetii* isolation, cultivation and purification

The isolate names, geographical/sample origin, plasmid type and associated clinical disease are listed in Table S1. *C. burnetii* were grown at 35°C on L929 cells using MEM (GIBCO, Invitrogen, Cergy-Pontoise, France) supplemented with 4% SVF (GIBCO) and 1% L-glutamine (GIBCO). Monolayers of cells and the supernatants from three 175 cm^2^ flasks were harvested and incubated with 1% trypsin (GIBCO) for 1 hour at 37°C. Released bacteria were purified from L929 cell debris by differential centrifugation. Purified bacteria were resuspended in 400 µl of PBS and stored at −80°C.

### gDNA extraction and amplification

Two hundred microliters of purified bacteria were incubated for 30 min at 70°C with 200 µl of AL lysis buffer (Qiagen, Courtaboeuf, France) and 20 µl of proteinase K (Qiagen). gDNA was extracted and purified using a QiaAmp DNA mini kit as recommended by the manufacturer (Qiagen). gDNA purity and concentration was checked using a NanoDrop (Thermo, Wingmilton, USA). Subsequently, 10 ng of gDNA were amplified with the processive polymerase phi29 using the GenomiPhi illustrator V2 kit (GE HealthCare, Lifescience, Orsay, France). This strategy was previously described for CGH experiments [14].

### gDNA labeling and microarray experiments

The amplified gDNA was labeled with the Bioprime CGH Labeling kit (Invitrogen) using d-CTP Cy3/5 fluorochromes (GE HealthCare Lifescience) as recommended by the manufacturer. Labeled amplified gDNA was purified using Pure Link PCR purification columns (Invitrogen), and the level of fluorochrome incorporation was quantified using a NanoDrop. Hybridizations were carried out using two samples of labeled amplified gDNA (150 pmol of each) that were labeled with Cy3 or Cy5 d-CTP. The pooled samples were hybridized using the GE hybridization kit (Agilent Technologies) as recommended by the manufacturer. The mixture was applied to a Surhyb 1 array (Agilent Technologies) and hybridized on the *Coxiella burnetii* array using an Agilent hybridization chamber (Agilent Technologies). Microarrays were hybridized for 17 h at 62°C in a rotating oven. Microarrays were washed using GE washing buffers (Agilent Technologies), with 5 min of Wash-buffer 1 at room temperature, followed by 1 min of Wash-buffer 2 at 37°C. Microarrays were dried using an acetonitrile bath (VWR, Fontenay sous Bois, France) and scanned using a microarray scanner C (Agilent Technologies) with XDR at a 5-µm resolution.

### *Coxiella burnetii* whole-genome microarray construction

OligoArray 2.0 [27], [28] was used to design probes from 2,016 CDSs extracted from the NC_002971.gb Genbank sequence file corresponding the genomic sequence of the Nine Mile reference strain without plasmid [3]. OligoArray 2.0 integrates a BLAST analysis against a non-redundant set of sequences and probe secondary structure analyses [29]. Oligonucleotide calculation parameters were set as follows: oligo length from 50- to 52 mers; GC percentage from 35% to 55%; melting temperature from 82°C to 86°C. OligoArray 2.0 selected probes with the lowest cross-hybridization and an absence of secondary structure and balanced the set of probes in terms of melting temperature. Oligonucleotides containing five consecutive As, Cs, Gs or Ts were discarded. Following probe design, 1990 probes corresponding to 1990 distinct CDS where selected for synthesis. Probes were ordered from Sigma-Proligo (Paris, FRANCE) as 5′ amino-modified oligonucleotides. Oligonucleotide stocks were aliquoted for use in microarray fabrication. Oligonucleotides were diluted to a final concentration of 35–50 µM in 35% dimethyl sulfoxide (DMSO) containing 100 mM potassium phosphate (pH 8.0). *Coxiella burnetii* - 2 k microarrays were printed with a ChipWriterProarrayer (Bio-Rad Hercules, CA) on commercial HydroGel slides (Schott, Mainz, Germany) and processed according to the manufacturer's instructions. Our microarrays were spotted in quadruplicate and contained 1990 different probe genes, corresponding to ca. 98.7% of ORF and around 5% of the total coding sequence excepting plasmid ORF. The microarray design have been deposited in the GEO database (http://www.ncbi.nlm.nih.gov/geo/) under GEO platform accession number (GPL6675).

### Analysis of microarray data

All microarray results have been deposited in the GEO database (http://www.ncbi.nlm.nih.gov/geo/) under GEO series accession number (GSE31543). The signal intensity and local background were measured for each spot using the array pictures with Feature Extractor software (Agilent Technologies). Data filtering normalizations were obtained using processing signal from obtained data raw extraction using Feature Extractor. We used the means of four replicates per probe to construct an M-A plot. Using the M-A plots, we deduced a naïve cut-off [30] to obtain genes that are putatively lost or highly divergent from our reference. A matrix for clusterization of our data was constructed using 0 for conserved genes and 1 for genes that were putatively lost or highly divergent. Clustering analyses were performed using Tmev [31], [32]. We used hierarchical clustering to generate a dendrogram with Euclidean distance and complete linkage for distance metric calculation and linkage methods, respectively.

### Genomotyping and statistical analysis

To perform genomotyping, we identified the putative single events of mutation. A matrix for clusterization of our data was constructed using 0 for conserved genomic content and 1 for putative events of mutation. Clustering analyses were performed using Tmev [31], [32]. We used hierarchical clustering to generate a dendrogram with Euclidean distance and complete linkage for distance metric calculation and linkage method, respectively. Statistical analysis was performed using GraphPad Prism5 (GraphPad Software, Inc.). A principal components analysis (PCA) was performed using Comprehensive Meta-analysis software (Biostat, Englewood NJ).

## Results

### CGH experiments

A CGH experiment using a whole-genome microarray was performed to genomotype 52 isolates of *C. burnetii* to detect deleted genes, as compared to the reference strain Nine Mile chromosome sequence (NC_002971). The information from the collection of isolates is listed in Table S1. To confirm gene losses, we compared the results to the previous study using CGH in *C. burnetii* [14]. We found comparable results between our deleted gene set and the set obtained by the previous CGH study. The strains used in both studies (HzS and S217) present similar gene content (Table S2). Given the putative gene losses deduced from the hybridization data from the 52 isolates, the genomic content *of C. burnetii* appears highly conserved across the 52 tested isolates (Table S1). The chromosomal deletion associated with phase II conversion [33] was found in only two isolates (HzR and Luga) and will not be included in this study. Comparative analysis showed that relative to the NMI strain, the percentage of deleted or highly divergent ORFs ranged from 0 – 2.5 % in S217. Heat map visualization of genomic variations showed that differences are spread across the genome (Figure S1). Only 161 genes from the NMI isolate were predicted to be absent or highly divergent in at least one tested strain (Table S3). Clustering analysis of genes that were putatively deleted in at least one isolate indicated that there were three distinct clusters (clusters 1, 2 and 3) (Figure S2). Clusters 1 and 2 contained genes with a high deletion frequency, whereas cluster 3 was mostly composed of 95 genes with a globally low deletion frequency. Hot spots of variation are observed along the chromosome (Figure S3).

### Genomotyping

**Figure 1:**
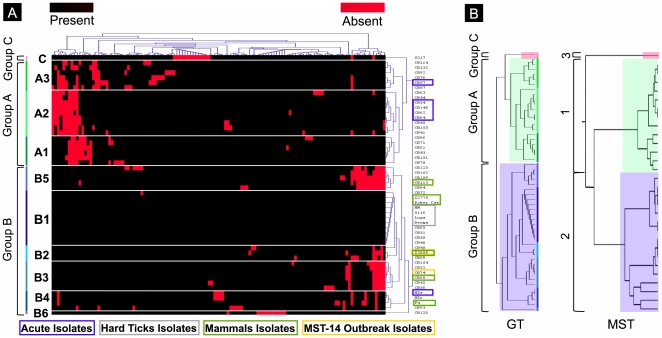
Typing of the collected isolates. (A) Genomic content clusterization of the isolates based on mutational events that allow determining different genomotypes. (B) Comparison of topology for genomotyping and MST typing.

To perform genomotyping, we first identified deleted or highly divergent genes and assigned them as single chromosomal mutation events (Table S4 and Table S5) based on the methods described by Beare *et al.* [14]. As shown in Figure 1A, we found that the isolates were organized in two major (A and B) and one minor (C) group that contained respectively 21, 30 and 1 isolate(s). Groups A and B were comprised of 3 (A1 to A3) and 6 (B1 to B6) distinct genomotypes, respectively. Low variability of genomic content was observable within the genomotypes. However, only a few isolates exhibited identical gene content, and small divergences occurred within genomotypes. We also found that group A was associated with deleted gene cluster 1 while clusters 2 and 3 were associated with groups A, B and C.

### MST-typing and genomotyping

We compared our genomotyping results to the MST genotyping results described previously [13], which described 3 groups presenting a similar topology (Figure 1B). MST genotypes 1 to 10 were included within group 1. Genotype 21 was included within group 2, and the other MST genotypes were associated with group 3 Thus, genomotype groups A, C and B include respectively MST genotypes 1 – 8, MST genotype 21 and the other MST-genotypes. We found only three exceptions in this analysis, as isolates CB76, CB93 and CB94 were not associated with the expected genomotype groups. Despite these exceptions, Figure 1B shows that there was low divergence between the genomotyping and MST genotyping results, and the two different methods showed congruence in the clusterization of isolates.

### Gene content and genomotyping associated with acute infection

We attempted to find genes associated with the acute clinical form of Q fever. We found that 4 clusters were associated with acute infection (A2, A3, B4 and B5). We hypothesized that these 4 clusters represented genomotypes that may cause acute infection. Thus, we focused on genes that were specifically deleted in acute isolates and their clusters and were present in chronic clusters and *vice versa*. We found that 4 deleted ORFs were significantly associated with acute infection isolates but also genomotypes containing acute infection isolates and their clusters (Table 1). These genes are annotated as hypothetical protein.

**Table 1:** ORFs associated with acute infections.

Locus Tag	Description	Acute isolates (7)	Acute Genomotypes Isolates (20)	Other (32)
CBU_1214	Hypothetical protein	5	13	0
CBU_1216	Hypothetical protein	5	13	0
CBU_1215	Hypothetical protein	6	16	0
CBU_0563	Hypothetical protein	7	17	0

The table shows the number of isolate presenting the putative deletion of different ORFs. We performed the investigation for 3 different categories, the isolates associated with acute manifestation (Acute isolates), isolates of the genomotypes that contain isolates associated with acute manifestation (Acute genomotype Isolates) and isolates from genomotypes that do not contain isolates from associated with acute manifestation.

### Gene content and genomotyping associated with physiopathology

**Figure 2:**
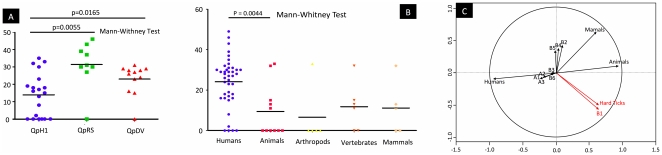
Association with gene repertoires and information. (A) Association of plasmid type and the number of deleted genes. (B) Association of the source of isolation and the number of deleted genes. (C) Representation of PCA analysis of source and genomotyping data. The blue circle represents the strongest associations.

We sought to determine whether the gene content of different strains could be correlated with the physiological and geographical information listed in Table S1. Comparisons of these data showed that the human isolates appeared to have more genes deleted than the animal isolates (Figure 2A). Furthermore, the arthropod isolates presented fewer deleted genes, particularly from the hard ticks (Luga, Derma, 5116 and NM), which did not present any deleted or highly divergent genes (genomotype B1). We also found that isolates associated with the plasmid QpH1 has fewer deleted genes compared to those isolates from the plasmids QpRS and QpDV (Figure 2B). Animal isolates were also principally associated with the QpH1 plasmid type (Table S1). We performed PCA to detect associations between gene absences or genomotypes and clinical or geographical information. We did not find any obvious associations with gene absence, but we did confirm, as previously mentioned, that the B1 genomotype is associated with arthropods, particularly with hard ticks (Figure 2C).

## Discussion

In this study, we examined the genomic content of 52 isolates of *C. burnetii* compared to the reference strain NMI using a global genomic approach based on comparative genomic hybridization by whole-genome microarray. A previous CGH analysis of *C. burnetii* was performed on 23 different isolates and two antigenic variants of NMI. Our collection included 3 strains in common with those used by Beare et *al*. (NMI, S217 and HzS), which were used as positive controls in this study. Although microarray used in the previous study has many more probes (covering approximately 30% of coding) compared to our home-made microarray (5% of coding sequence), we found high homologies between our results and those of Beare et al. [14] (Figure S1). In this regard, we conclude that we obtained robust and confirmed data to perform genomotyping with our microarray results. Several typing methods have been developed for the causative agent of Q fever [5], [7], [8], [10], [12]–[14]. Glazunova et *al.* [13] and Beare et *al.* [14] showed that these different methods of typing are globally congruent. In our study, we compared whole-genome typing with MST methods. This comparison showed that the two methods produced a similar clusterization of isolates within three groups with significantly divergent gene content (Figure 1B). Thus, the different isolates of *C. burnetii* present divergent evolution of the three groups that is independent of geographic origin and clinical context, as previously proposed by Glazunova et *al.* [13] and Beare et *al.* [14].

**Figure 3:**
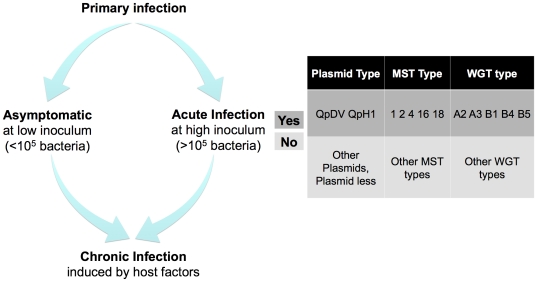
Bacterial factors involved in Q fever.

In several animal models, both the amount of inoculum used and the strain influence the presence and manifestations of acute pneumonia during Q fever [17], [18], [34]. However, there is no evidence that isolates from chronic and acute human infections differ when large collections are screened by different methods of typing [13]. A preliminary analysis based on MST-typing showed that acute Q fever was induced by isolates belonging to MST genotypes 1, 2, 4, 16 and 18 and that the plasmid QpDV was highly associated with acute Q fever. Isolates from chronic infections were associated with all MST-genotypes and all of the observed plasmid types. In our study, we found that only genomotypes A2, A3, B4 and B5 contained isolates from acute infections. Based on these associations, we found four deleted or highly divergent ORFs with unknown functions that were significantly associated with acute Q fever. Furthermore, the previous microarray genomic analysis showed that isolates from acute infections had a comparable gene repertoire to that of genomotype B1 [14]. Because no genomotype was specifically associated with chronic Q fever, we confirmed that all isolates could be involved in chronic infections, as previously proposed [13], [16]. Previously, Beare *et al.* has mainly associated acute Q fever with B1 genomotype [14]. We described four additional genomotypes that were associated with acute infections compared to the previous study. Based on the results obtained using previous methods of typing collected isolates and on studies of acute Q fever in animal models, we have schematically represented the putative factors involved in Q fever infections (Figure 3). After the primary infection, we speculate that minimal inoculum is necessary to induce acute manifestations in case of the strain present specific plasmids, MST genotypes or genomotypes. The chronic manifestation could be induced by all the strains whether the host presents favorable clinical field.

A bias in sampling exists in this study. Whereas chronic Q fever affects 20 times fewer patients than the acute form of the disease, most of the human isolates used here were from chronic disease patients, and the isolates from acute infections were mainly obtained from France. However, our collection of isolates contained 2 isolates presenting an identical MST-genotype to the putative epidemic clones, which came from a patient suffering from chronic Q fever in Marseille (CB74) and from the placenta of a goat in Germany (Z3055). The 2 isolates revealed comparable, but different gene repertoires and were associated with different genomotypes.

Surprisingly, the comparative genomic analysis of our isolate collection revealed that animal isolates (especially from arthropods) exhibited fewer deleted genes globally than human isolates (Figure 2A). All isolates from hard ticks presented gene content identical to the Nine Mile I strain. Beare et *al.* [14] previously found that two isolates associated with hard ticks exhibited identical gene content to NMI (Dugway 5G61-63 and BDT 1), and *C. burnetii* has been identified in many species of ticks [26].

A major limitation of our study is that we only used the genome of the reference strain Nine Mile to design the microarray [3]. It was the only genome available at the beginning of our study, and the addition of sequences from other strains may contribute to a better understanding of the *C. burnetii* cycle and Q fever pathophysiology. However, we found here, for the first time, that isolates from hard ticks exhibit the same gene content and probably the same origin. Moreover, we observed the loss of 4 putative genes associated with virulence, fuelling the hypothesis that bacterial pathogenicity is driven more by gene loss than gene gain.

## Supporting Information

Figure S1 **Representation of genomic content of the 52 isolates.** The figure represents the genomic variation of the different isolated compare to the reference strain Nine Mile I. The red marked ORFs are considered as deleted and the black marked ORFs are considered as conserved.
(PDF)

Figure S2 **Clusterization of genes deleted at least one time among the collected isolates.** The figure represents a hierarchical clustering of the genomic content among isolates. The red marked ORFs are considered as deleted and the black marked ORFs are considered as conserved. The hierarchical clustering has been performed using the average linkage and the Euclidian distance for classification of isolates and ORFs both.
(PDF)

Figure S3 **Frequency of gene deletions.** The figure represents the frequency of variation that could occur within the different isolates along the Nine Mile I chromosome. The frequency along the chromosome is represents as heat map (A) and as histogram (B).
(PDF)

Table S1 ***C. burnetii*** **isolates used in this study.** * Epidemic genotype isolates.
(XLSX)

Table S2 **Comparison with the two different CGH studies.** The table represents the comparison of results obtained from the two CGH studies. Grey cells represent genes that are not spotted. Black cells represent ORFs that have not been found deleted. Red cells represent ORFs that have been found deleted. Orange cells represent ORFs that have been found partially deleted. Bleu cells represent ORFs with a small insertion
(XLS)

Table S3 **Genomic content of the 52 isolates.** The table presents a matrix with the ORFs (with annotation) that have found deleted in at least 1 isolate and the different strains (with information). In the matrix the value 0 is associated to non-deleted ORFs and the value 1 to deleted ORFs.
(XLS)

Table S4 **Different genomic events found.** The table presents the different events assumed in our study. Yellow cells represent ORF that have been found deleted in our study and the event associated with a combination of deletion is written in blue.
(XLS)

Table S5 **Genomic events found in the 52 isolates.** The table presents a matrix with genomic events that have been assumed for each strain. In the matrix the value 0 is associated to the absence of the genomic event and the value 1 to the presence of the genomic event.
(XLS)
