# Supplementary figures and images for: Genomotyping of Coxiella burnetii Using Microarrays Reveals a Conserved Genomotype for Hard Tick Isolates
Source: PLoS One. 2011 Oct 25;6(10):e25781. doi: 10.1371/journal.pone.0025781 (PMC3201959; doi:10.1371/journal.pone.0025781)

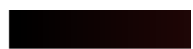

Present

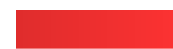

Deleted

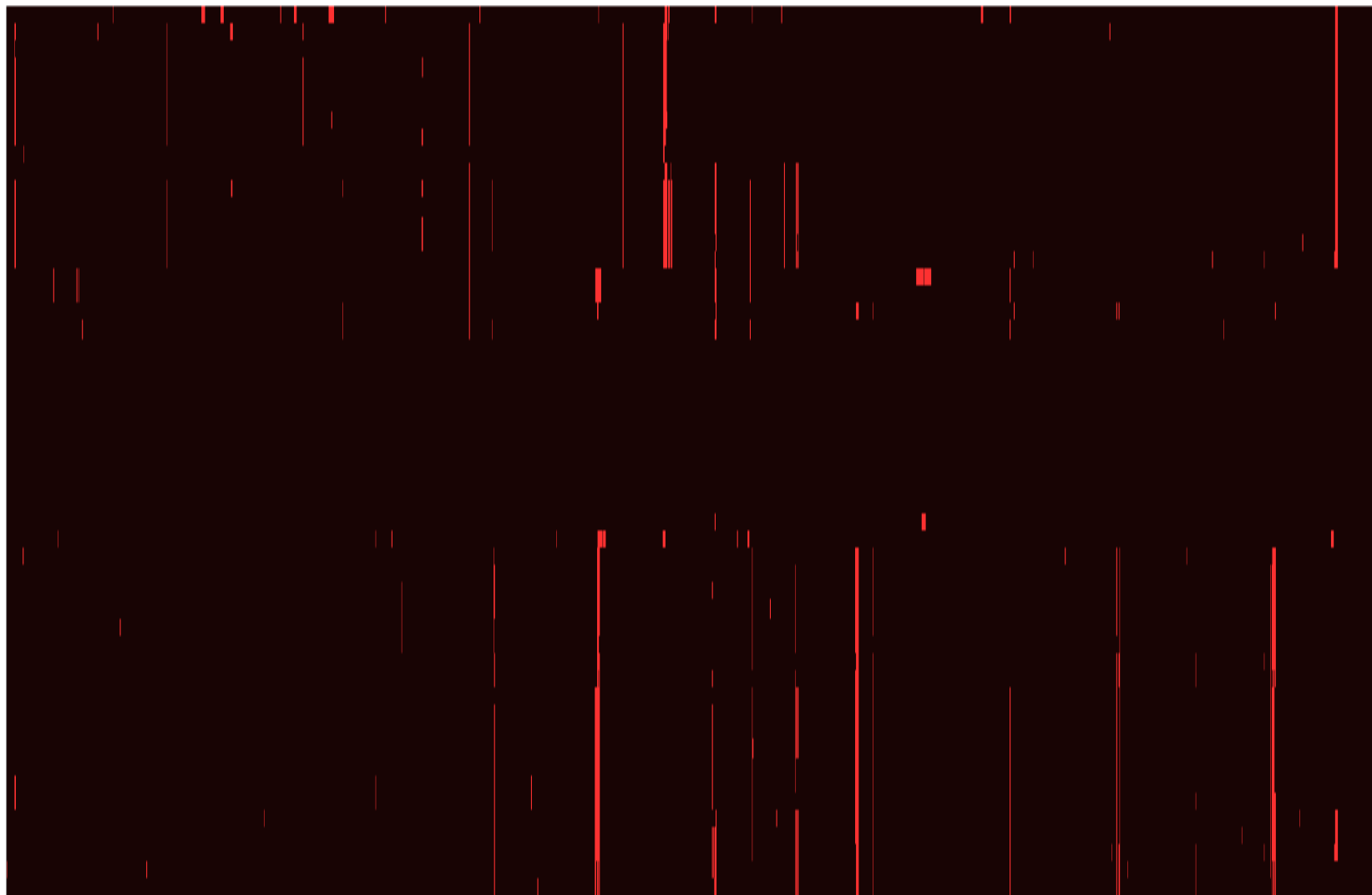

Supplement: Figure S1 — Representation of genomic content of the 52 isolates. The figure represents the genomic variation of the different isolated compare to the reference strain Nine Mile I. The red marked ORFs are considered as deleted and the black marked ORFs are considered as conserved. (PDF) [file pone.0025781.s001.pdf]

Present

Deleted

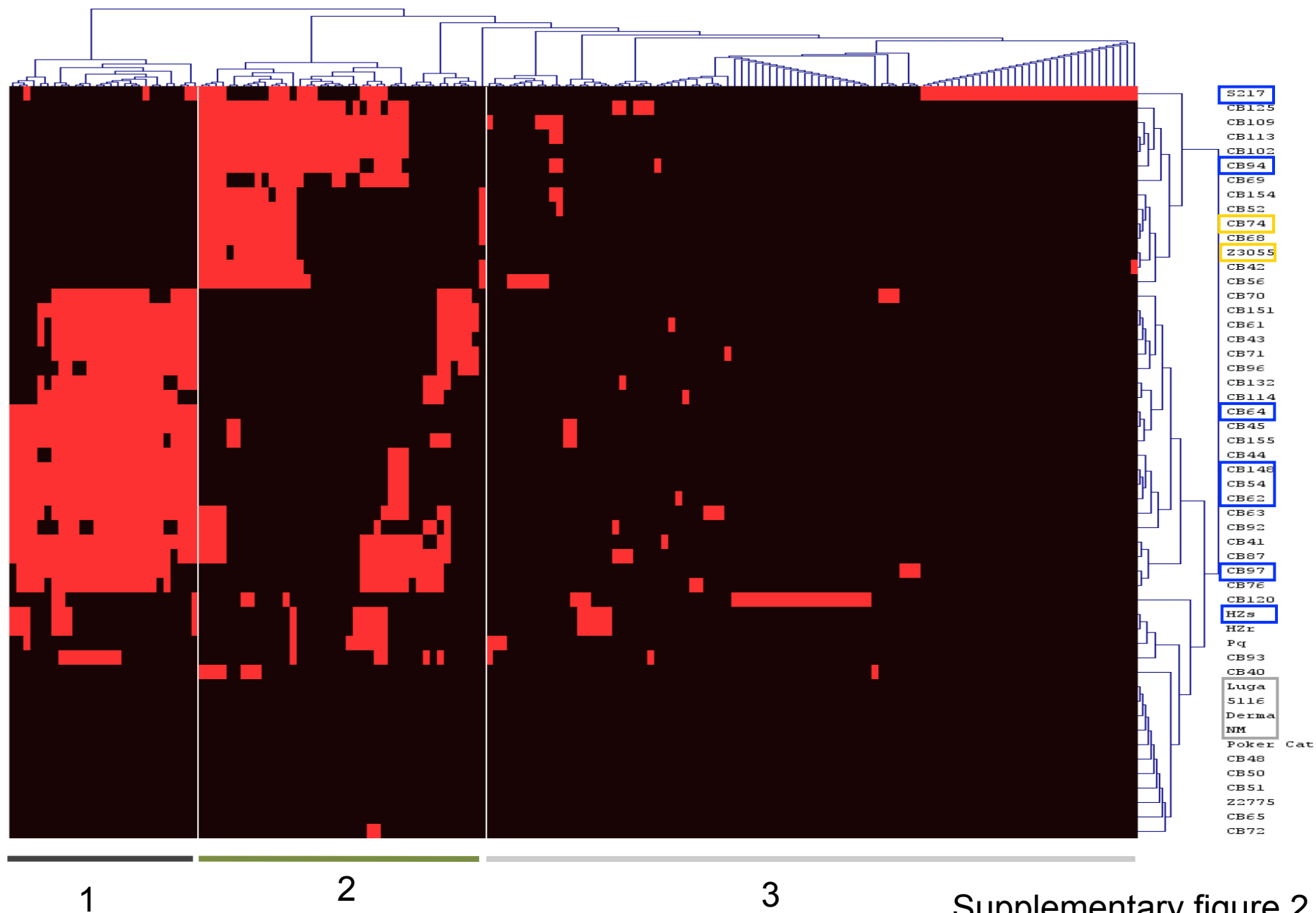

Supplement: Figure S2 — Clusterization of genes deleted at least one time among the collected isolates. The figure represents a hierarchical clustering of the genomic content among isolates. The red marked ORFs are considered as deleted and the black marked ORFs are considered as conserved. The hierarchical clustering has been performed using the average linkage and the Euclidian distance for classification of isolates and ORFs both. (PDF) [file pone.0025781.s002.pdf]

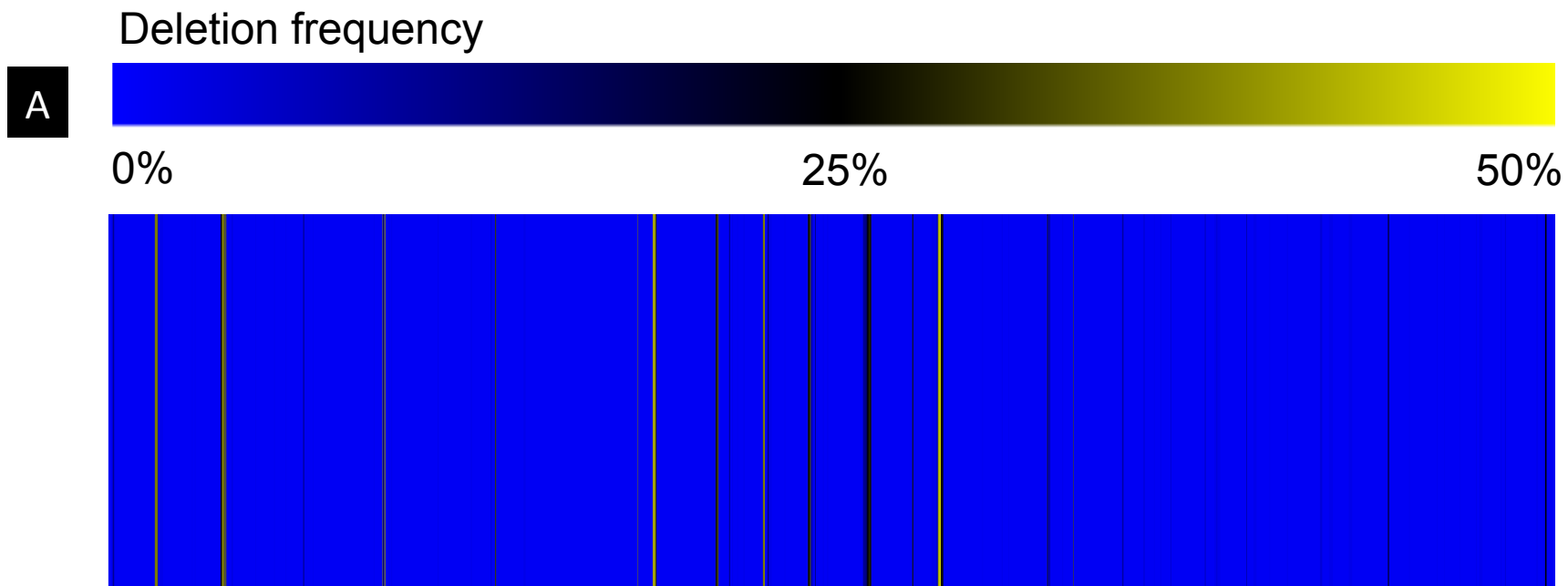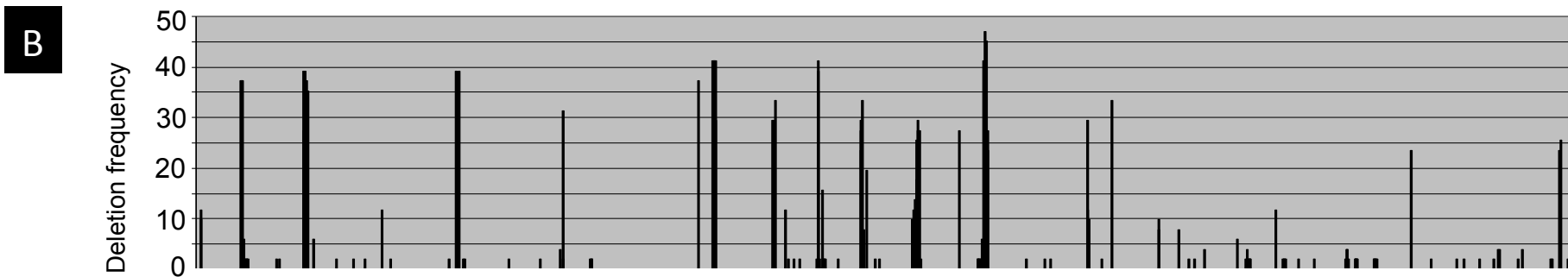

Supplement: Figure S3 — Frequency of gene deletions. The figure represents the frequency of variation that could occur within the different isolates along the Nine Mile I chromosome. The frequency along the chromosome is represents as heat map (A) and as histogram (B). (PDF) [file pone.0025781.s003.pdf]
